# Juvenile Osteochondritis Dissecans: Current Concepts

**DOI:** 10.7759/cureus.65496

**Published:** 2024-07-27

**Authors:** Ibrahim Akkawi, Hassan Zmerly, Maurizio Draghetti, Lamberto Felli

**Affiliations:** 1 Orthopedics, Casa di Cura Villa Erbosa, Bologna, ITA; 2 Orthopedics, Villa Erbosa Hospital, Bologna, ITA; 3 Orthopedics, San Martino Hospital, Genova, ITA

**Keywords:** autologous adipose-derived mesenchymal stem cell implantation, autologous chondrocyte implantation, autologous osteochondral transfer, microfracture, drilling, juvenile osteochondritis dissecans

## Abstract

Osteochondritis dissecans (OCD) primarily damages the subchondral bone, leading to damage to the articular cartilage. Juvenile OCD (JOCD) of the knee is limited to skeletally immature and young patients with open growth plates on radiographs. We conducted a review of PubMed articles up until March 16, 2024, using a combination of the following keywords: knee, juvenile, and osteochondritis dissecans. This narrative review included a total of 56 relevant articles that investigated the etiology, incidence, clinical presentation, imaging, classification, and treatment of JOCD of the knee in patients less than 20 years of age. The exact etiology is controversial. Most authors believe that the disease involves multiple theories, such as ischemia, recurrent trauma, and genetic predisposition. Radiographs, the first imaging study in this patient group, cannot determine the stability or instability of the surface cartilage of the OCD lesion. As a result, MRI has become a recommended diagnostic method for determining OCD stability and providing important information for determining a treatment plan. For stable JOCD lesions, nonsurgical treatment is often advised. For unstable and stable lesions that do not respond to nonsurgical treatment, several surgical techniques with good healing rates are available.

## Introduction and background

Osteochondritis dissecans (OCD) is believed to predominantly impact subchondral bone, leading to further damage to the articular cartilage [1]. The most frequently affected part is the knee [2]. Juvenile OCD (JOCD) is a condition that affects skeletally immature patients and has visible growth plates on their X-rays [3]. Based on the histological cartilage study [4], 20 years of age is considered the transitional age between adult and juvenile cartilage, meaning that patients with JOCD are those who are younger than 20 years at the time of presentation. The majority of the reviews in the literature included studies that addressed JOCD of the knee in patients over 20 years of age [5] and addressed the only surgical outcome of JOCD without mentioning the outcome of conservative treatment [6]. To the best of the authors’ knowledge, no review has addressed the new surgical techniques for treating JOCD of the knee, such as the use of cell-free osteochondral collagen scaffolds [7], one-step bone marrow-derived cell transplantation [8], and autologous adipose-derived mesenchymal stem cell (AD-MSCs) implantation [9]. Hence, this narrative review seeks to provide an updated report on the literature regarding the etiology, incidence, clinical presentation, imaging, classification, and conservative and surgical treatment, with a focus on new surgical techniques for knee JOCD in patients less than 20 years of age.

## Review

Methods

A comprehensive search of the PubMed database was performed until March 16, 2024, utilizing a combination of the keywords “knee,” “juvenile,” and “osteochondritis dissecans.” In order to ensure that no relevant documents were missed, a comprehensive analysis was conducted on each reference list from the selected research. The inclusion criteria consisted of studies conducted in English that focused on patients with JOCD of the knee who were under the age of 20. The exclusion criteria encompassed studies conducted in languages other than English, research focusing on individuals above the age of 20 with OCD, and studies specifically addressing JOCD in joints other than the one under investigation.

Results

Out of the total of 231 papers found in the original search, 175 were excluded since they did not match the specified inclusion criteria. This narrative review includes a comprehensive analysis of 56 relevant papers [1-56] that examine the etiology, incidence, clinical presentation, imaging, classification, and management of JOCD in individuals under the age of 20.

Discussion

Etiology

The precise origins of it are mostly unclear. The prevailing consensus among experts is that the condition encompasses several theories, including repeated trauma, ischemia, genetic susceptibility, and anatomical causes [10-12]. As a result of repeated trauma, the subchondral bone is believed to develop a stress fracture, leading to necrosis and detachment of the bone from the articular cartilage that surrounds it [13]. Inflammatory diseases [10], being overweight [14], having a lateral discoid meniscus [15], undergoing meniscal surgery for a juvenile discoid lateral meniscus [16], having a narrower intercondylar notch width [17], having a posterior cruciate ligament insertion further distally on the femur in patients with medial femoral condyle (MFC) OCD compared to patients with lateral femoral condyle (LFC) OCD and normal knees [18], having medial and lateral tibiofemoral subluxation [19], and having leg malalignment [20] may also cause OCD. Jacobi et al. [20] have shown a correlation between varus alignment and medial condyle lesions, as well as between valgus alignment and lateral condyle lesions. Additional morphological features in individuals with MFC OCD lesions include a higher degree of posterior and medial tibial slope [21], a more noticeable tibial eminence [22], and increased mobility of the anterior horn of the meniscus [23].

A retrospective investigation [24] revealed a surprisingly high incidence of vitamin D deficiency among individuals first diagnosed with JOCD, suggesting a potential association between vitamin D deficiency and the development of JOCD. Hussain et al. [25] found a potential connection between hGH insufficiency, hormone replacement therapy, and the development of OCD lesions.

Incidence

Based on a recent survey, knee JOCD is ranked as the fourth most common injury among young athletes [26]. The majority of patients engage in activities such as baseball, football, and basketball, which involve significant axial loading and jumping [10,26,27]. The incidence is higher among males compared to girls [10]. According to retrospective research [28], the prevalence of OCD in children between the ages of 6 and 19 is 15.4 per 100,000 for men and 3.3 per 100,000 for girls. Furthermore, the prevalence of OCD in the 12-19 age group was 3.3 times higher compared to the 6-11 age group.

In recent years, the rising participation of young children, regardless of gender, in competitive sports has influenced the prevalence of this illness. Thus, there is evidence to suggest that the average age at which JOCD becomes apparent is decreasing, and it is more common among females [29]. According to several authors, the real prevalence and incidence of a condition may be underestimated due to patients without symptoms and incorrect diagnoses made during clinical evaluations [3]. The MFC is the joint most frequently afflicted by OCD of the knee, accounting for 69% of cases. The LFC follows at 15%, the patella at 5%, and the femoral trochlea at 1% [30].

Clinical Assessment

Based on the studies conducted by D’Angelo et al. [3] and Kumar et al. [10], it is often observed that patients usually suffer from pain and stiffness during physical activity. These symptoms are aggravated by periods of severe exercise or activities, such as climbing hills or stairs. In addition, there may be sporadic instances of clicking, locking, and catching, which suggest the presence of unstable lesions or loose intraarticular bodies.

Evaluation may reveal joint effusion in 23-44% of patients and point pain on the affected femoral condyle in 40-70% of patients [2]. Only advanced or prolonged lesions show atrophy of the quadriceps muscles of the affected limb [3]. During the initial phases of the disease, the knee’s ability to move is often normal. However, as the condition progresses and loose bodies emerge, pain or mechanical blockage may restrict the knee’s range of motion to passive extension [3]. Stable lesions or a loose body may induce crepitus during the knee range of motion [13]. Both stable and unstable lesions can result in an antalgic gait [29]. Extending the knee while internally twisting the tibia (known as the Wilson sign) can cause pain. However, in a sample of 32 patients who had OCD lesions in the MFC, the Wilson sign was insensitive, as 75% of the tests yielded negative results [31]. Certain individuals may experience difficulty walking, such as the need to externally rotate the tibia to avoid the tibial spine from coming into contact with the lateral side of the MFC [2].

Imaging

For patients with a clinical history and physical examination results consistent with JOCD, radiographs serve as the initial imaging assessment to assess lesion healing [30]. Radiographic evaluations of patients with knee OCD generally include anteroposterior, lateral, tunnel, merchant, and sunrise views [2].

A clearly defined subchondral bone region, split by a radiolucent contour in the shape of a crescent, characterizes an OCD lesion on radiographs. Nevertheless, the presence of subchondral irregularities or lucencies in one or both femoral condyles may indicate normal growth in people who are still developing their skeletal structure. This phenomenon is generally referred to as developmental ossification variation or irregular ossification [30]. Moreover, radiographs are unable to determine the stability or instability of the surface cartilage in an OCD lesion, a critical factor in selecting an appropriate treatment plan [5]. Frequent observations have revealed disparities between the data acquired by radiography and that gained during surgery. For instance, fragments that seem to have detached from their docking location on X-rays remain secure and protected by healthy cartilage during arthroscopic surgery [32]. As a result, MRI has become more important as the preferred diagnostic technique for assessing OCD. In addition, it tracks the progression and recovery of OCD, assists in identifying the likely site of the lesion, and assesses whether the lesion is stable or unstable [32,33].

De Smet et al. [34] first outlined the MRI criteria for assessing the instability of OCD lesions, which include the existence of a high signal intensity between the OCD lesion and nearby bone; a joint fracture indicated by the passage of high signal joint fluid through the subchondral bone plate; a localized defect in the bone and cartilage filled with joint fluid; or a cyst filled with fluid that is at least five millimeters deep within the lesion. Despite its high sensitivity (97-100%), the specificity of this MRI finding is limited (11-55%), particularly in patients who are still growing. [2]. Heywood et al. [35] found that there was a 30% agreement between the MRI and arthroscopy phases, as evaluated by Dipaola et al. [36]. Roßbach et al. [33] used arthroscopy as a standard to test how well preoperative MRI could diagnose OCD of the knee and talus in children. They employed the OCD classification system of Dipaola et al. [36] and found that the total accuracy of MRI was just 41.3%. In addition, Samora et al. [37] found that preoperative MRI had limited accuracy in identifying JOCD. There was only a 62.1% agreement between Dipaola et al.’s MRI [36] and Guhl’s arthroscopic grading [38].

According to O’Connor et al. [39], unstable lesions are defined as those with a disruption in the articular cartilage on T1 and a high signal intensity on T2 located behind the fragment. This refinement significantly improved MRI’s accuracy in assessing the stability of JOCD lesions, raising it from 45% to 85%. In addition, Kijowski et al. suggested a novel MRI classification approach specifically tailored for JOCD [40]. In patients with JOCD, the presence of three specific signs indicates 100% accuracy in diagnosing lesion instability. These signs include a JOCD lesion surrounded by a high T2 signal intensity border that has the same signal intensity as the nearby joint fluid, a secondary outer border with low T2 signal intensity, and multiple breaks in the subchondral bone plate. In addition, they found that perilesional cysts larger than 5 mm in diameter (with a sensitivity of 25% and a specificity of 100%) or several cysts (with a sensitivity of 38% and a specificity of 100%) were also indicators of instability.

Both patients who underwent surgery and those who received conservative therapy commonly underwent MRI scans to assess their recovery progress [33]. The MRI results, primarily visible on non-fat-suppressed T1-weighted or GRE-weighted images, reveal that the healing process following nonoperative treatment entails a decrease or elimination of the surrounding bone marrow edema, a reduction in the lesion’s size, a decrease or elimination of the intense signal border or cyst-like areas, and the growth of bone within the OCD lesion, which connects to the nearby subchondral bone [30].

Classification

While plain radiography is commonly used to assess healing progress, it is not the most effective technique for determining the severity of lesions because it does not offer a comprehensive evaluation of the articular cartilage. MRI can provide a more accurate classification of JOCD lesions. The MRI classifications most commonly utilized are Hefti et al. [41] and Dipaola et al. [36]. The arthroscopic classifications most commonly utilized are Guhl’s [38], the International Cartilage Repair Society’s [42], and Ewing and Voto’s [43].

Nonoperative Treatment

Stable JOCD lesions with intact articular cartilage frequently require nonoperative treatment [27]. Experts recommend a minimum of three to six months of nonoperative treatment [33]. Nonoperative methods such as immobilization and activity restriction often treat stable lesions in skeletally immature patients. A brace, cast, or standard knee immobilizer can achieve successful immobilization [29]. Research studies have shown that prolonged immobilization might have negative effects on the knee’s functionality [2]. During a period of six to eight weeks, those undergoing nonoperative treatment should refrain from participating in sports and engaging in high-impact activities. However, for patients who provide their agreement, it is possible to resume regular weight-bearing activities [44]. However, if the patient continues to experience symptoms during routine activities such as walking, we gradually increase their weight-bearing limits until they achieve asymptomatic status [13]. During the initial three months, it is recommended to engage in other forms of exercise such as swimming, stationary cycling, deep-water running, and other activities that have less impact on the body. It is advisable to engage in regular activities and sports after a period of around four to six months [3,10].

Reportedly, the success rate of nonoperative therapy in this specific group of patients varies between 49% and 95% [1,45,46]. Krause et al. [45] found that after one year of nonoperative therapy, 49% of patients with JOCD either fully healed or were in the process of healing. Hughes et al. [46] found a 95% recovery rate over a five-year period in 21 knees affected by JOCD. The discrepancies in results may be attributed to the many MRI instability criteria utilized by writers, each having a unique level of specificity and sensitivity.

Research has shown that the location and size of a lesion have an impact on its ability to recover. Hefti et al. [41] found that lesions with a diameter bigger than 20 mm had a worse prognosis, as shown by a significant multicenter study. Kumar et al. [10] found that lesions with smaller longitudinal diameters and surface areas, as identified by MRI, have a higher likelihood of healing without the need for surgery. Furthermore, lesions located in atypical areas have a noticeable deficiency in their ability to recover. Vannini et al. [8] discovered that the magnitude of the lesion had a detrimental impact on the extent of improvement in the clinical result. In their retrospective cohort examination of 37 patients treated surgically for knee JOCD, Bangert et al. [12] found that defects with a depth of less than 0.8 cm^2 ^resulted in significantly better outcomes compared to defects with a depth of 0.8 cm^2^ or larger. The location, size, and BMI at the time of surgery did not have an impact on the outcome. Patients with JOCD who had a discoid meniscus and a longer duration between onset and consultation had a worse outcome when treated nonoperatively in the LFC [1].

Operative Treatment

A range of surgical techniques are used for unstable JOCD of the knee as well as for stable lesions that do not heal with nonoperative therapy, with healing success rates of 62%, according to a systematic review by Eismann et al. [5]. Trinh et al. [6] conducted a review that showed that surgical treatment for knee OCD in young patients significantly enhances clinical and radiological outcomes in the short, medium, and long term. Their analysis determined that, when comparing many surgical procedures, there were no discernible disparities in clinical or radiographic results.

Reparative procedures include transarticular and retroarticular drilling [47], reduction and fixation [48-50], and microfracture [51]. Autologous osteochondral transfer, autologous chondrocyte implantation (ACI), cell-free osteochondral collagen scaffold, one-step bone marrow-derived cell transplantation, and AD-MSCs implantation are examples of restorative treatments.

Transarticular and retroarticular drilling of the subchondral bone can be employed to treat stable lesions in individuals who have not shown improvement with nonoperative therapy. The objective is to facilitate the recovery of JOCD lesions that are less than 2.5 cm^2^ [10,30]. In order to stimulate the regrowth of blood vessels and the healing of the damaged subchondral bone, vascular paths are created using the bone marrow underneath it [30]. Gunton et al. [47] conducted a comprehensive analysis of the short-term clinical outcomes of drilling stable JOCD lesions. The study found that there were no notable disparities in the rates of radiographic healing for JOCD lesions treated with retroarticular or transarticular drilling procedures. The healing rates were 86% and 91%, respectively.

Various techniques of internal fixation utilizing diverse metallic implants have been employed to treat OCD lesions [49]. Complications related to these implants include the necessity for subsequent surgery to remove them, interference with MRI scans, insufficient compression, fracture of the pins, loosening of the implants, and migration through the skin [48,56]. Due to these complexities, bioabsorbable implants have been created. Regrettably, due to their inability to compress, bioabsorbable implants have been associated with negative outcomes, such as synovitis and loss of fixation [49]. Webb et al. [48] conducted a retrospective review of 20 patients who had internal fixation of their unstable JOCD lesions using either bioabsorbable fixation, metal fixation, or a combination of both. The patients were followed up clinically for an average of seven years. During the final evaluation, 15 out of 20 knees (75%) showed osseous integration when assessed by radiographic imaging. Tabaddor et al. [49] conducted a retrospective case series to assess the outcomes of patients with unstable JOCD who were treated with bioabsorbable pins. The study included twenty-four knees, and the patients were followed up for an average of 39.6 months. The MRI scan showed that 15 out of 17 patients (88.2%) experienced either partial or complete recovery, while 22 out of 24 patients (91.7%) had favorable to outstanding clinical results. Similarly, in a study conducted by Komnos et al. [50], it was shown that 90% of patients with JOCD of the knee who had retrograde drilling and arthroscopic stabilization of the lesion with bioabsorbable pins showed evidence of lesion healing based on MRI data. Out of the 40 patients included in the study, 36 showed successful healing.

Microfracturing and OAT procedures are conducted to address defects that are less than 2-3.5 cm^2^ [12,51]. The suggested technique of microfracture entails creating several microscopic perforations in the subchondral bone with the aim of attracting stem cells and growth factors to the damaged cartilage area. Gudas et al. [51] conducted a clinical trial where they randomly assigned participants and observed them over a period of 4.2 years. They found that 63% of the 23 patients who had microfracture surgery for the treatment of JOCD anomalies in the femoral condyles of the knee joint experienced excellent or satisfactory clinical results. In the study conducted by the same authors [51], it was shown that 83% of the patients with JOCD (19 out of 23) had excellent or good clinical results after undergoing OAT. The average follow-up period was 4.2 years. Furthermore, 91% of the patients, namely 19 out of 21, exhibited good or adequate repairs as shown on MRI scans. In 11 patients, Miniaci and Tytherleigh-Strong [52] reported the results of using OAT to treat symptomatic, unstable JOCD lesions. After six months of surgery, they found that repeated MRI scans showed complete healing of the osseous component of the lesion in all knees. Similarly, Miura et al. [53] discovered that none of the six cases of JOCD lesions treated with OAT showed contact between the osteochondral fragment and subchondral bone on MRI three months after surgery.

ACI is often the preferred method for defects larger than 3 cm^2^ [12]. Beck et al. [4] conducted longitudinal cohort research to determine the long-term, patient-based outcomes of ACI treatment for knee JOCD in 10 patients. They discovered a failure rate of 20% over an average period of 12 years. The study conducted by Moriya et al. [54] found that six individuals with knee JOCD who underwent ACI treatment experienced similar results. The ultimate follow-up demonstrated an 85% success rate. In a study conducted by Krishnan et al. [55], it was shown that after an average follow-up period of 4.3 years, 82.1% of patients had excellent or good outcomes, and 96.4% of the 28 patients with JOCD of the knee had successful results.

In a study conducted by Sessa et al. [7], the researchers evaluated the results of a new method for treating knee JOCD in 20 patients. The methodology involved employing a collagen scaffold that did not contain any cells. Upon the final follow-up, it was seen that all scores exhibited notable enhancement, and MRI scans indicated complete filling or slight enlargement of the cartilage defect in 84.6% of the patients. Vannini et al. [8] monitored six patients with knee JOCD who had a treatment involving the transplantation of bone marrow-derived cells, platelet-rich plasma, bone marrow concentrate, and a hyaluronic acid scaffold in a single step. They showed that, in general, the patients saw a considerable decrease in symptoms at their final follow-up, enabling them to resume the majority of their everyday activities and sports. Furthermore, the newly formed tissue, assessed by MRI, completely occupied the whole volume of the osteochondral defects in every instance. According to a recent prospective study conducted by Russo et al. [9], a 13-year-old patient with JOCD of the patella experienced pain relief and improved function after one year of follow-up. Additionally, there was an improvement in cartilage volume and the structure of the injured area after two years of follow-up, thanks to the use of AD-MSCs.

## Conclusions

Knee JOCD typically affects young and growing people. It is believed to predominantly harm the subchondral bone, leading to further damage to the articular cartilage. The precise cause of this condition is not known; however, several possibilities have been suggested, including repeated trauma, ischemia, hereditary predisposition, and anatomical variables. While radiographs are typically the initial imaging examination conducted for these individuals, they are unable to ascertain the stability or instability of the surface cartilage in the JOCD lesion. Consequently, MRI has gained importance as the preferred diagnostic technique for assessing the stability information of JOCD, which is crucial for establishing a treatment strategy. Conservative treatment is often advised for stable JOCD lesions with intact articular cartilage. Various surgical procedures are available for the treatment of stable lesions that do not heal with nonoperative therapy and unstable knee JOCD, with high percentages of successful healing.
